# Mini-Mental State Examination among lower educational levels and illiterates: Transcultural evaluation

**DOI:** 10.1590/S1980-57642010DN40200008

**Published:** 2010

**Authors:** Sonia Maria Dozzi Brucki, Ricardo Nitrini

**Affiliations:** 1 MD, Behavioral and Cognitive Neurology Unit, Department of Neurology, University of São Paulo School of Medicine, São Paulo SP, Brazil.

**Keywords:** Mini-Mental State Examination, illiteracy, educational level, screening test

## Abstract

Cognitive performance among illiterates and low educational levels is poorer than that observed in individuals with greater schooling. This difference can be a confounding factor in reaching an accurate diagnosis of cognitive impairment. In addition, there is great heterogeneity in performance among illiterates, probably due to different environmental demands and sociocultural backgrounds. Many reports have described the influence of education on neuropsychological measures and screening tests such as the Mini-Mental State Examination (MMSE).

**Objectives:**

To analyze performance in two samples with the same educational level, but different social and cultural backgrounds.

**Methods:**

Subjects from two different locations in Brazil (rural sample from Northern region and urban sample residing in the largest city of the Southeastern region) were matched for age and education, and submitted to the MMSE.

**Results:**

Significant differences between the groups were found in total scores on the MMSE and in temporal orientation and serial-sevens sub-items for which the urban sample performed best but analysis of illiterates alone yielded the same results, except for the copying pentagons task which was performed better by the rural sample.

**Conclusions:**

Cultural and social backgrounds, as well as demands from the environment, influence results of screening tests. Factors other than education must be taken into account when analyzing tests.

## Abstract (pt)

O desempenho cognitivo entre iletrados e indivíduos com baixos níveis de escolaridade é pior do que observado entre indivíduos bem escolarizados. Esta diferença pode ser um fator de confusão quando um diagnóstico preciso de comprometimento cognitivo se faz necessário. Além disto, há uma grande heterogeneidade no desempenho destas pessoas, provavelmente devido a diferentes demandas ambientais, e base sociocultural. Muitos estudos tem descrito a influência educacional em medidas neuropsicológicas, bem como em testes de rastreio, como o Mini-Exame do Estado Mental (MEEM).

**Objetivos:**

Analisar o desempenho de duas amostras de mesmo nível educacional e diferentes bases sociais e culturais.

**Métodos:**

Indivíduos de dois locais no Brasil foram submetidos ao MEEM, pareados por idade e escolaridade.

**Resultados:**

Houve diferença significativa entre as amostras quanto ao escore total e sub-itens de orientação temporal e sete-seriados, com melhor desempenho para a amostra urbana, mas quando analisados somente os analfabetos, os mesmos resultados foram observados, exceto para a cópia dos pentágonos, onde a população rural teve desempenho melhor.

**Conclusões:**

A base social e cultural além das demandas do meio ambiente influenciam nos testes de rastreio. Outros fatores, além da educação, devem ser revistos quando analisarmos os testes.

Cognitive performance among illiterates and low educational levels is poorer than that observed in individuals with greater schooling. This difference can be a confounding factor in reaching an accurate diagnosis of cognitive impairment. In addition, there is great heterogeneity in performance among illiterates, probably due to different environmental demands and sociocultural backgrounds. The theory of cognitive reserve seeks to explain these findings, since more cognitive demands due to place and activities are factors stimulating brain development. In Brazil, many reports have described the influence of education on neuropsychological measures. Screening tests such as the Mini-Mental State Examination are influenced by schooling, with different cut-off scores. Other cognitive batteries show the same effect, with significance difference among scores by literacy and educational level, as observed on the Dementia Rating Scale,^1^ CERAD cognitive battery^2^ and ADAS-Cog.^3^ Surprisingly, some tests, apparently free of the influence of education, such as Luria’s fist-edge-palm, immediate memory, naming simple drawings are also affected by education. Illiterates possibly do not have an usual grapheme-phoneme association, hence their learning and retrieval of information via the semantic pathway,^4^ which could be an important difference in illiterates from different backgrounds. Adaptation and validation of tests in each country and in different environments are crucial to minimize the false-positive rate for cognitive impairment in low educated people. Another important point is the concept of functional illiteracy, which can cause a bias in cognitive performance. Cognitive measures are effective for providing diagnostic clues in higher educated subjects, but are unable to achieve this in illiterates or functional illiterates. Norms are necessary for low educated individuals, and neuropsychological tests should be adapted and extensively applied to this group in order to define their cognitive profile.

Since the devising and publication of the Mini-Mental State Examination (MMSE) by Folstein et al.^5^ many studies have been published about the influence of educational levels on scores. The MMSE is commonly used in Brazil as a screening test for cognitive evaluation and as an instrument to follow-up demented patients. This test has become widely used as a screening tool for cognitive impairment worldwide, with numerous translations and adaptations. It was recommended by the Scientific Department of Cognitive Neurology and Aging of the Brazilian Academy of Neurology as a screening test for Alzheimer’s disease.^6^ Almost immediately, researchers perceived a bias in scores in relation to educational level.^7-9^ Lower educated individuals presented different cut-off scores. Similarly, Anthony et al.^10^ described that the sensitivity of the test was lower in African Americans than in White patients, and that this difference could be due to an educational bias, while specificity was also found to be lower in subjects with less than 8 years of schooling. Other authors have studied low educated populations along with other peculiarities such as cultural background and race, but the common factor linking results for differences among samples was educational level.^11,12^ More recently, Rosselli et al.^13^ carried out a population-based survey involving a random sample of urban and rural residents in Colombia. In this study, the MMSE scores correlated with educational level, where its effect was greater than that of age or gender. These authors concluded that low specificity led to the identification of many nondemented subjects with low educational status requiring further investigation.

Many reports have shown similar effects in Brazil, with different scores according to schooling.^14-19^

Performance of illiterates and lower educational groups have interested and led our group to study the influence of the cultural and natural environment on some tests. To this end, we compared people residing in the largest city of São Paulo (a highly industrialized city), Southeast Brazil, to those residing in the Amazonian region (Mamirauá Sustainable Development Reserve - MSDR), North of Brazil. There are many differences between these two locations, besides geographical and environmental differences. For instance, subjects from the Northern region are more likely to have native Indian roots and consequently different sociocultural habits and values^20^ to those of inhabitants of a Southern city. These differences allowed comparison of individuals with the same educational levels but different cultural backgrounds for a widely used screening cognitive test, the MMSE. We expected that with the greater environmental demands of a big industrialized city, subjects from São Paulo would outperform Northerners on scores.

## Methods

### Subjects and environmental setting

A sample of subjects residing in the Mamirauá Sustainable Development Reserve (MSDR) was matched by education to subjects residing in São Paulo. All subjects were Portuguese-speakers and part of a larger sample evaluated in the region. Participants included those present in the MSDR at the time of the research team’s visit, aged 50 years or over, who were given a thorough medical examination. Clinical, neurological, neuropsychological and anthropometric evaluations were carried out. For this report only results on MMSE test are presented. The visits were made by boat to the MSDR over seven periods of 12 to 15 days each, comprising single visits for periods of one or two days per community. Subjects were interviewed at their homes or in community centers. Subjects were interviewed on the day of the research team’s arrival.

**Figure 1:**
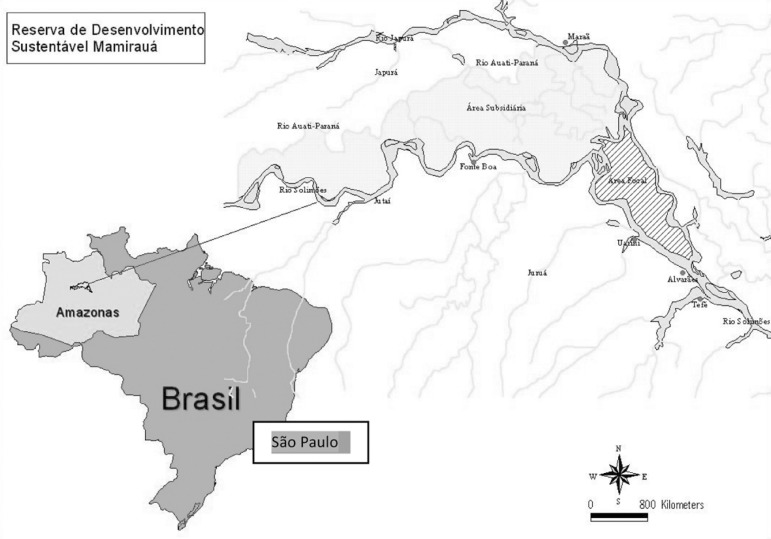
Mamirauá Sustainable Development Reserve and São Paulo City.

The MSDR is located about 600 km West of Manaus (Amazonas), in the Brazilian Amazonian region (Figure 1). These are conservation units designed to combine the preservation of habitats with the sustainable development of local resident communities. These areas are seasonally flooded, with water levels rising 10 to 12 meters above regular levels. The MSDR had 5,615 inhabitants, 435 of whom were over 49 years old (7.7%)(data provided by the Mamirauá Institute, census of 2002). The population lives in small communities along the river banks, each with 13 domestic households on average, where these are typically linked by kinship ties characterizing them as nuclei of small groups of related people. It is essentially a subsistence-based economy with very low incomes (annual family incomes of about US$900). Activities comprise fishing, growing manioc for flour and in some communities, hunting. The houses are timber-built and elevated from the ground because of high water levels. Activities extend throughout the lifetimes of most subjects. Elderly individuals were the only group presenting reduced physical activities. Women tended to work making flour, housekeeping and taking care of children. Almost all communities have schools, providing education for up to four years. The communities have limited access to radio, and television broadcasting, newspapers, daily use of telephone, and bank accounts. Electricity is produced by diesel generator (when available). The state of health is considered good, but there is a great difficulty in accessing health services which entails travelling to other urban centers, sometimes involving a 12 to 18-hour trip by small motor boat. The resident population has little access to formal education. Of the total population older than 15 years of age, 38% is illiterate while the others have an educational level of up to 4 years. Furthermore, the majority of the population is exposed to the same influences and opportunities of educational increments. Reading habits were scarce, generally limited to the Bible or reading in the classroom by the school children. Many adults that reported receiving several years of formal education were unable to read simple sentences.

We evaluated 65 inhabitants aged 50 and older, based on collection of demographic data as well as a self-evaluation of subjects’ reading, writing and capacity to sign. Individuals were considered illiterate when they fulfilled all of the following three conditions: they had never attended school, or had attended for less than one year; they considered themselves unable to read, and were unable to read the phrase “close your eyes” from the Mini-Mental State Examination. None of the subjects were taking medications with central nervous system action.

Participants were matched by educational level, gender, and age (within a five-year range) to subjects without cognitive complaints and independent daily activities, spouses or caregivers of patients of the Neurology outpatient clinic in Hospital Santa Marcelina (HSM). This hospital is located in a suburb of the Eastern region of São Paulo city; it is responsible for healthcare assistance of about three million people, predominantly subjects with lower educational level and socioeconomic conditions (C, D, and E classes - ABIPEME). Clinical evaluation was not performed in this sample.

Exclusion criteria for both samples were motor difficulties, neurological or psychiatric complaints, and uncorrected visual or hearing deficits.

### Evaluation

All subjects answered a semi-structured questionnaire about general conditions and subsequently performed the Mini-Mental State Exam (MMSE). For the MSDR sample, we provided some modifications in spatial orientation due to local peculiarities, and therefore the items became: name of the community, name of the city, nearest city, nearest community, and State, replacing the original items: street or county, specific place, general place.^15^ The remaining items were as per the Brazilian version. For the HSM sample all items of the version of Brucki et al were used.^15^

### Statistical analyses

For statistical analyses, comparisons involving the MSDR and HSM samples were carried out using the Student t- test. The significance level was 0.05. The Statistica 4.3 (Statsoft, Inc, 1993) program was used for the analyses.

The survey was approved by the Research Ethics Committee of the Hospital das Clínicas of the São Paulo University School of Medicine, of Hospital Santa Marcelina, and of the Mamirauá Institute. All subjects had given written consent for their participation in the study, or relatives had given written consent on behalf of those subjects unable to sign.

## Results

**Figure 2:**
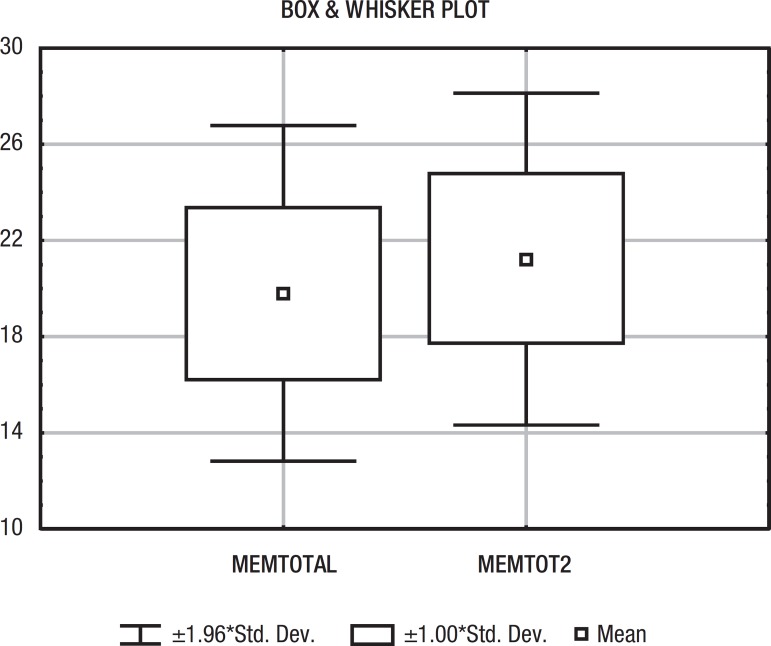
Means on MMSE. MEMTOTAL, MSDR sample; MEMTOT2, sample of HSM.

Overall, there were 37 females in the samples (56.9%), and in the MSDR sample, 84.6% of subjects performed subsistence agriculture activities, while subjects in the São Paulo sample carried out a variety of activities. There was no difference between the two samples regarding age, schooling (Table 1) or gender. Results on the MMSE are shown in Table 1 and Figure 2. Analyzing only illiterates (n=42) from both samples, the copy of pentagons task was the only different result in relation to the full samples, on which the MSDR sample presented significantly better performance (Table 2), a pattern repeated in the full sample.

**Table 1:** Sample comparisons regarding age, schooling, and MMSE scores.

	MSDR (n=65)Means (SD)	HSM(n=65)Means (SD)	p-value
Age	50.1 (19.5)	52.5 (17.3)	0.448
Schooling	1.2 (1.7)	1.1 (1.8)	0.799
Total score	19.8 (3.6)	21.2 (3.5)	0.023
Temporal orientation	3.8 (1.2)	4.5 (0.7)	0.003
Spatial orientation	4.2 (0.9)	4.3 (0.9)	0.601
Immediate memory	2.9 (0.2)	2.9 (0.2)	0.812
Serial "sevens"	0.4 (1.2)	1.5 (1.4)	<0.001
Recall	1.8 (1.0)	1.9 (1.1)	0.603
Naming	2.0 (0.17)	2.0 (0.0)	0.299
Repetition	0.8 (0.4)	0.8 (0.4)	0.732
Command	2.9 (0.3)	2.9 (0.4)	0.299
Reading	0.3 (0.5)	0.3 (0.4)	0.599
Writing	0.3 (0.4)	0.2 (0.4)	0.604
Copy of pentagons	0.4 (0.5)	0.2 (0.4)	0.063

* t-test.

**Table 2:** Sample comparisons regarding age, and MMSE scores among illiterates.

	MSDR(n=42)Means (SD)	HSM (n=42)Means (SD)	p value*
Age	58.1 (19.0)	51.9 (16.4)	0.331
Total score	18.2 (2.8)	19.6 (2.7)	0.024
Temporal orientation	3.5 (1.3)	4.4 (0.7)	0.005
Spatial orientation	4.0 (0.9)	4.3 (0.9)	0.246
Immediate memory	2.9 (0.2)	2.9 (0.2)	0.632
Serial "sevens"	0.02 (0.2)	1.1 (1.2)	<0.001
Recall	1.7 (1.1)	1.9 (1.1)	0.627
Naming	2.0 (0.2)	2.0 (0.0)	0.284
Repetition	0.8 (0.4)	0.8 (0.4)	0.646
Command	2.9 (0.3)	2.8 (0.4)	0.204
Copy of pentagons	0.3 (0.5)	0.04 (0.2)	0.001

## Discussion

Our results showed a statistically significant difference between the samples in total scores on the MMSE, temporal orientation and calculation, where subjects of São Paulo city performed better than those of Mamirauá. In contrast, illiterates from Mamirauá outperformed subjects from São Paulo on the copy of pentagons task, perhaps possessing better visuospatial perception than the illiterates from São Paulo city. Although there was no significant difference in age, the MSDR sample was older and thus aging-related perceptual deficits could not explain this finding. Samples were also matched by gender, minimizing possible gender differences. In developing countries such as Brazil, we have some difficulty classifying an examined illiterate because of different social, economic, and cultural backgrounds. Although some adaptations were made in spatial orientation for the MSDR subjects on the MMSE, no performance difference was detected on this sub-item. In fact, only items that are more closely related to environmental demands such as temporal orientation, and serial sevens and that seem more important for those living in a big city, such as making changes, or obeying strict schedules may explain this difference. We could not explain this difference as a function of other differences between samples, because the samples were matched by gender, and educational levels.

Several studies have described comparisons among heterogeneous populations on MMSE scores, and emphasized the need to review specific differences.^21-25^ Some differences in sub items analysis are due to language group differences, such as those described in a report comparing English and Spanish-speaking subjects, in which discrepant items were orientation to season, state, repeat phrase, and follow command.^26^

A number of Brazilian studies have shown median or mean scores for illiterates to be very analogous considering this is a pool of subjects with different performances, reporting median scores of 18;^14^ 20;^15^ and 19^16^ points, and a mean of 17.4 (4.0).^18^ However, in a large sample, Kochhann et al.^19^ observed no differences between illiterates and schooled subjects, but noted a major impact of education among different educational groups.

In clinical practice, we observed heterogeneous performances for the same conditions of illiteracy. This subjective impression is confirmed by systematic evaluation. Analyzing 79 healthy illiterates (between 26 to 82 years of age) and their performance on MMSE, and then dividing them into two groups based on lower quartile (P25=16 points) and upper quartile (P75=20 points), Brucki noted a significant difference in temporal orientation (year, semester, day of the week), spatial orientation (floor), calculation, copy of pentagons and reading. No differences were detected in naming, repetition or verbal command.^27^ This study proved that many other factors probably influence illiterate performance besides education, including social and cultural factors. Previous history and occupations in life are important aspects to observe because they demand varying requirements and cognitive effort.

We can hypothesize that the cultural influences and environmental demands a person experiences represent an influencing factor. It is likely that in the larger cities, even illiterates are better able to cope with some formal demands made in test situations. Indeed, when we homogenized samples by excluding literates, the differences were found to persist.

In this study, we demonstrated that environmental and other sociocultural aspects are very important in cognitive performance, besides education, among individuals from the same country and with the same language. Other surveys should be performed on samples from various regions of Brazil to determine specific differences by geographical area.
